# Words of Encouragement

**Published:** 1915-07

**Authors:** 


### Words of Encouragement.

Dr. Ira S. Wile, the editor of *Medical Review of Reviews,* published the following complimentary notice in his May issue:

"In this issue, dedicated to the women physicians of America, we wish to make mention of the fact that the TEXAS MEDICAL

JOURNAL is published and edited by a woman. Mrs. F. E. Daniel, the editor, has made a notable success of this journal, and we wish to compliment and encourage her for the valuable periodical which she is building up.

"The TEXAS MEDICAL JOURNAL devotes a great deal of space to the question of eugenics, and its Motherhood Department is unique. Women physicians would undoubtedly be very much interested in the journal, a sample copy of which will be sent upon request, by addressing Mrs. Daniel at Austin, Texas."

---

It is very gratifying to note that you are maintaining the high standard of the TEXAS MEDICAL JOURNAL. I sincerely hope that your splendid publication may be continued for many years to come.

With kindest regards and best wishes, I am,

Sincerely yours,

JORDAN W. LAMBERT,
St. Louis, Mo.

---

I am enclosing my check for $1.00, the price of your TEXAS MEDICAL JOURNAL, and am pleased to say that the last issue was indeed a "hummer."

A. L. ANDERSON,
Brownwood, Texas.

---

*Dear Mrs. Daniel:*

Enclosed herewith find postoffice money order for $1.00 for my subscription to the "Red Back." I take great pleasure in reading it. I am a subscriber from the start; I took No. 1, and have read every number since.

Yours sincerely,

W. M. AUSTIN, M. D.

---

*Dear Mrs. Daniel:*

The May number of your journal is just received, and I have looked it over with considerable interest. You certainly have managed to publish a very attractive journal, and I am glad to see that your friends, the advertisers, still remain loyal to you. The friends your husband made many years ago were evidently the right kind.

We are very glad that the publication comes out so attractively every month, as it must be a source of much satisfaction to you. With kindest personal regards, I am,

Yours very sincerely,

THE PURDUE FREDERICK COMPANY,

Albert E. Stratton, President.

